# Using Trust to Establish a Secure Routing Model in Cognitive Radio Network

**DOI:** 10.1371/journal.pone.0139326

**Published:** 2015-09-30

**Authors:** Guanghua Zhang, Zhenguo Chen, Liqin Tian, Dongwen Zhang

**Affiliations:** 1 State Key Lab of Integrated Service Networks, Xidian University, Xi'an, Shannxi, China; 2 College of Information Science and Engineering, Hebei University of Science and Technology, Shijiazhuang, Hebei, China; 3 School of Information Science & Engineering,Northeastern University,Shenyang, Liaoning, China; 4 College of Computer Science and Technology, North China Institute of Science and Technology, East Yanjiao,Beijing, China; Beihang University, CHINA

## Abstract

Specific to the selective forwarding attack on routing in cognitive radio network, this paper proposes a trust-based secure routing model. Through monitoring nodes’ forwarding behaviors, trusts of nodes are constructed to identify malicious nodes. In consideration of that routing selection-based model must be closely collaborative with spectrum allocation, a route request piggybacking available spectrum opportunities is sent to non-malicious nodes. In the routing decision phase, nodes’ trusts are used to construct available path trusts and delay measurement is combined for making routing decisions. At the same time, according to the trust classification, different responses are made specific to their service requests. By adopting stricter punishment on malicious behaviors from non-trusted nodes, the cooperation of nodes in routing can be stimulated. Simulation results and analysis indicate that this model has good performance in network throughput and end-to-end delay under the selective forwarding attack.

## Introduction

The shortage of wireless spectrum resources is the bottleneck of the sustainable development of wireless mobile communication and service application. As an intelligent revolutionary spectrum sharing technology, CR (cognitive radio) [1] is regarded as the hottest future wireless technology. It not only can perceive SOPs (spectrum opportunities) [2], but also can adjust transmitting power, operating frequency and other parameters according to the dynamic changes of the environment, enabling CR terminals to opportunistically work in licensed frequency bands. At the same time, the access of non-licensed users cannot interfere with users’ communication in a licensed frequency band. In this way, the optimal frequency spectrum utilization can be realized. CR terminal interconnection forms CRNs (cognitive radio networks) [3] and it introduces cognitive characteristics into the whole wireless communication network, which can distinguish the current network states. Then according to these states plans, decisions and responses are made and the knowledge from the self-adaptive process can be used to make pre-decision for end-to-end performance.

CR terminals constitute nodes of CRNs, aiming at completing the perceptive function of the physical layer. CRNs mainly cover the technology of all the layers in the communication network, including network layer and transport layer and other high-layer technology. Current studies mainly focus on the problems of CRNs physical layer and MAC layer [3, 4]. Some optimal solutions suitable for single-hop topological structure are proposed. However, they are not suitable for multi-hop CRNs. In the multi-hop communication of CRNs, how to quickly and accurately select route from source end to destination node is an important research task. Dynamic changes of node SOPs make it necessary to apply a cross-layer design idea to the route design in CRNs, namely, routing selection of the network layer must be closely collaborative with spectrum allocation in MAC layer. This has been successively put forward in relevant research results [5~11]. In Literature [5], traditional on-demand routing discovery is combined with spectrum sensing and each node maintains available SOPs list. Destination nodes select an optimal route according to throughput and SOPs list in request information is used to select an available channel in each hop. At the same time, data transmission nodes periodically broadcast channel reserved information to reduce the interference of data flow. In Literature [6], PSA (path spectrum availability) is defined according to the throughput between neighboring nodes and nodes select the next hop for data forwarding according to PSA and local SOPs. In Literature [7], RREQ (route request) and RREP (route reply) are sent through CCC (common control channel) and available SOPs information of each node is packaged in RREQ. Destination nodes select channels according to the minimum principle of path accumulating delay and package them in RREP. Specific to QoS in CRNs route, energy consumption control to routing solution is introduced in Literature [8]. In Literature [9], the bandwidth utilization is reduced by minimum spanning tree and time slot allocation algorithm. In Literature [10], bionics is used to optimize SOPs utilization and minimize the channel switching delay to improve the stability of CRNs route. In Literature [11], LEAVE messages are broadcast in the original channel firstly when nodes switch channels and JOIN messages are then broadcast in new channels, which is meant to avoid the problem of deafness. The above research work proposes the routing design and optimization plan specific to spectrum dynamic changes in cognitive radio networks, but the security threats of routing are not paid attention to. When nodes participating in routing forwarding maliciously discard data packets, the network availability decreases sharply.

Actually, distributed CRNs also are faced with attack behaviors [12] on routing in wireless sensor networks, for example, in selective forwarding attack malicious nodes make themselves in the path of data transmission through exchanging false routing information. They may refuse to transmit specific messages or directly discard some data packets, which seriously influences the throughput of networks, end-to-end delay and other overall performance. Traditional cryptography system-based security mechanism mainly is used to resist external attacks, but it cannot solve the above soft security threats caused by malicious nodes inside [13]. Trust management is considered as its efficient supplement. Trust management has been widely studied in peer-to-peer network and wireless sensor network. In peer-to-peer network, feedback-based trust system is propose in Literature [14], which utilizes feedback evaluation, transaction amount, feedback credibility, relevant factors with transaction and relevant factors with transaction environment to evaluate node credibility and describe various malicious behaviors, and it provides the distributed realization of this model. In Literature [15], credit and risk evaluation-based trust model is put forward, which considers the influence of nodes’ dynamic behaviors on the calculation of credibility and introduces risk factors and adopts information entropy theory to quantize risks, unifying the trust degree between entities and uncertainty of trust. In wireless sensor network, node credibility is used to show the reliability of sensing results and improve the application accuracy of target localization in Literature [16]. In Literature [17], the concept of functional trust is firstly come up with, which respectively constructs node trust according to node behaviors in different functions of sensing, data fusion and routing. Different trusts need different evaluation mechanisms. Based on node resource-constrained characteristics in wireless sensor networks, routes are selected through the trust demand of data packet, trust level of neighboring nodes and coverage range so as to improve the success rate of data forwarding and extend the life cycle of networks in Literature [18]. The above research work provides useful references to the study of trust mechanism in CRNs. Some research results apply trust mechanism in CRNs, for example, in collaborated spectrum sensing application, in Literature [19, 20] trusts are constructed through sensing results reported from nodes and further effectively identify malicious nodes and filter false data and enhance the robustness of fusion decision.

At present, there is no trust-based CRNs routing studies. This paper firstly introduces node trust evaluation into CRNs routing. In data forwarding phase, the behaviors of neighboring nodes are monitored for trust evaluation to defend the selective forwarding attack of malicious nodes. At the same time, trust-based CRN routing studies must take the availability of node SOPs into consideration. In CRNs, each node senses wireless spectrum and respectively decides the available and ever-changing SOPs. To highly-efficiently determine the next-hop in CRNs and communicate with it, it is inevitable to combine route selection with spectrum allocation. The paper effectively combines trust mechanism with CRN routing protocol in route discovery, route reply, route decision and route maintenance, and uses trust to guarantee the end-to-end data transmission. In route discovery phase, request messages only are forwarded to non-malicious nodes. After route returns, on the basis of nodes' trust of available paths, path trusts are calculated and combined with the delay measurement of paths for the selection of data forwarding paths. At the same time, according to node trust, reward and punishment mechanism is adopted to stimulate honest forwarding of nodes. The simulation experiment indicates that the route selection based on trust evaluation can effectively reduce the possibility of taking malicious nodes as the next hop so as to improve the stability of data transmission and obtain good network performance. The major contributions of this paper mainly manifest on three aspects as follows.

1. This paper firstly applies trust mechanism to solve the security problem in CRN route.
2. As one factor of route decision, path trusts are calculated according to node trust, which improves the reliability of data forwarding.
3. Reward and punishment mechanism is adopted to stimulate the collaboration of non-trust node in route.

### System Model

Reputation systems can be used to foster good behavior and to encourage adherence to contracts in e-commerce. Several reputation systems have been deployed in practical applications. The beta reputation system is based on using beta probability density functions to combine feedback and derive reputation ratings. The advantage of the beta reputation system is flexibility and simplicity as well as its foundation on the theory of statistics [21]. In distributed CRN topological structure, the beta reputation system^]^ is adopted to calculate node trust. Based on the characteristics of dynamic changes of node SOPs, in the routing model, trust mechanism is combined to enhance the robustness of CRN route under selective forwarding attack.

### Topological structure

**Fig 1:**
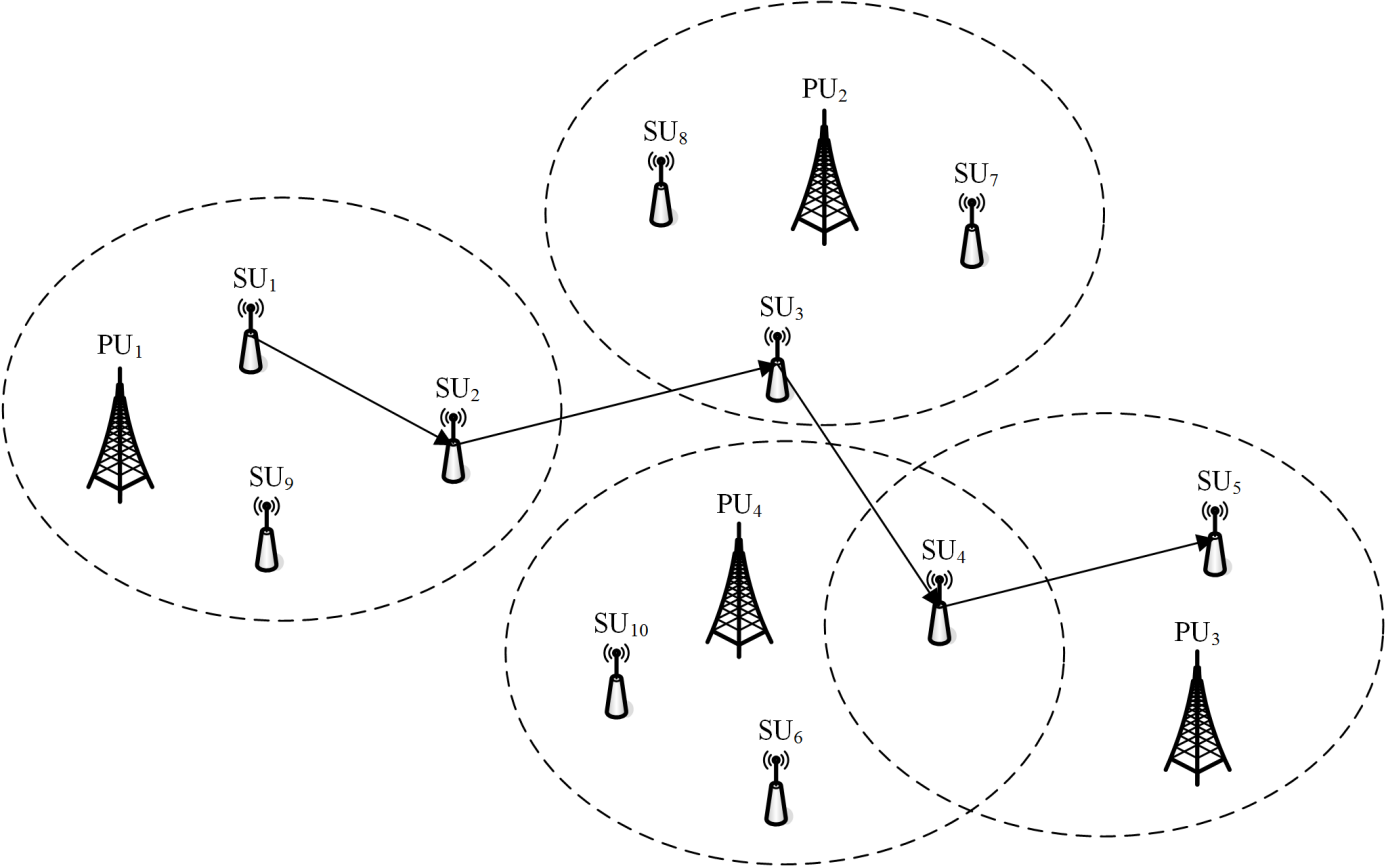
Topological structure of distributed CRNs.

CRNs are deployed in distributed type. The topological structure is shown as Fig 1. The important concepts are interpreted as follows.

1. There are two kinds of entities in networks, PU (primary user) and SU (secondary user). Primary users, also called licensed users, own licensed spectrum opportunities and are licensed to operate in specific frequency bands free from the influences of other users. Secondary users, also called unlicensed users or cognitive radio users, do not own any frequency band and continuously sense the licensed spectrum opportunities. It can self-adaptively adjust send-receive equipment to the currently sensed spectrum opportunity for communication in premise of guaranteeing the use of primary user in priority and no loss of transmission performance.
2. Spectrum opportunity, also called vacant spectrum or spectrum hole, is temporarily vacant spectrum in frequency, time and space, including unlicensed frequency and temporarily unused licensed frequency of primary users. If destination nodes are located outside of transmission range of source nodes and data packets must pass through the middle node to be routed to destination nodes. Namely, through multiple transmission, multi-hop CRNs forms. In Fig 1 there are 4 PUs and 10 SUs. The range of oval dotted line is PU effective area. SU uses SOPs communication. SU_1_ sends route data packets to SU_5_ through SU_2_, SU_3_ and SU_4_.
3. In multi-hop CRN route, middle nodes need to perceive SOPs and divide SOPs into multiple channels. Then, according to one strategy, one proper channel is selected to forward data packet. The neighboring nodes of communication must own the same available channel. Due to different location of primary users, SOPs detected by each node in CRNs may be different. Furthermore, because of the randomness of primary users, each node in CRNs needs periodic detection of SOPs. Thus, SOPs of the same node is not constant. In this paper, the existence of primary users is not considered, neither the route establishing of secondary users with primary users. The influence of primary users in route is abstracted into the influence of the node dynamic SOPs on route.

### Premise assumption

To guarantee that the transmission of routing information between SUs is free from the influence of different node SOPs, it is assumed that SU not only owns one cognitive wireless transceivers, but also owns one traditional wireless interface to form common control channel. Routing information and nodes’ SOPs information can be transmitted by common control channel. SUs can accurately and effectively perceive the changes of SOPs through certain spectrum sensing technology and they can share SOPs information at MAC layer and network layer based on the cross-layer design idea. At the same time, cognitive wireless transceivers of SU are assumed half duplex. At one moment, SU only can receive or transmit data at one channel, which is consistent with IEEE 802.11 terminal equipment. Multipath propagation and Doppler effects in wireless environment are major factors of channel changes. Multipath propagation is relevant to the surrounding wireless channel links. Doppler effects are relevant to carrier frequency and relative movement rate of transceivers. Influenced by the above factors, it is difficult to collect the real-time quality condition of channels. The focus of this paper lies in attack behaviors of malicious nodes under the same channel condition. Therefore, the throughput of each channel is assumed the same.

### Calculation of node trust

If there is no special instruction, secondary users are called nodes. In CRN route, node trust is defined as the reliability that nodes honestly forward data packets to the next hop. In centralized collaborated spectrum sensing, each SU reports the local sensing information to SU base station (also called fusion center) and SU base station is used for fusion decision of spectrum availability. To improve the accuracy of collaborated spectrum sensing, in Literature [20] nodes’ trust values are calculated according to whether the submitted sensing result of SU is true and by aid of the beta reputation system [21]. Nodes’ trusts are taken as the weight of local sensing results and false data provided by malicious nodes is filtered. However, this paper introduces trust mechanism into distributed CRN route and each node maintains channel information for the next hop to send data packet to monitor whether the next hop normally forwards data packets. This paper uses the statistics of node forwarding behaviors and the beta reputation system to construct the trust of neighboring node *j* from node *i*. In this way, malicious nodes can be identified and selective forwarding attack can be defended so as to enhance the robustness of route mechanism. Below is the detailed description of how to calculate the node trust by the beta reputation system in distributed CRNs.

The beta distribution usually is used to represent the posterior probability of one binary event. The beta distribution *f*(*p*|*α*, *β*) constituted of *α* and *β* can be expressed as Formula (1) in Γ function.

$$f(p|\alpha ,\beta )=\frac{\Gamma (\alpha +\beta )}{\Gamma (\alpha )\Gamma (\beta )}p^{\alpha -1}{\left(1-p\right)}^{\beta -1}\;0\leq p\leq 1,\;\alpha >0\;,\beta >0$$ (1)

Formula (1) satisfies the following constraint conditions, *p* ≠ 0 if *α* < 1 and *p* ≠ 1 if *β* < 1. The probability expectation of the beta distribution is shown as Formula (2).

E(p)=α/(α+β) (2)

For binary events with two results $\left\{x,\bar{x}\right\}$, r represents the occurrence times of result *x*; s represents the occurrence times of result $\bar{x}$. Through the setting in Formula (3), the occurrence probability density of result *x* of the current binary event can be expressed as the function of historical statistic results.

a=r+1,β=s+1r≥0,s≥0 (3)

In Formula (1), variable *p* represents the occurrence probability of result *x*; *f*(*p*|*α*, *β*) represents the probability of determined value of *p*. Because variable *p* is continuous, it makes no sense to calculate *f*(*p*|*α*, *β*) for given *p*. The only significance is to calculate the probability expectation of *p* in Formula (2).

In CRN route, the beta reputation system takes the behaviors of node forwarding data packet as binary event of the beta distribution modeling. From node *i*, if neighboring node *j* successfully forwards data packet to the next hop, it is noted as *x*_*ij*_; if not, it is noted as ${\bar{x}}_{ij}$. The times of successful forwarding and failed forwarding respectively are noted as *r*_*ij*_ and *s*_*ij*_. Trust *T*_*ij*_ of node *i* to neighboring node *j* represents the probability expectation that neighboring node forwards data packet to the next hop, shown as Formula (4).

$$T_{ij}=E(f(p|\alpha ,\beta ))=\frac{\alpha }{\alpha +\beta }=\frac{r_{ij}+1}{r_{ij}+s_{ij}+2}$$ (4)

Initially, *α* = *β* = 0, so *T*_*ij*_ = 0.5.

To give larger weight to the recent forwarding behaviors evaluation on nodes and gradually weaken the outdated evaluation information, time aging factor *λ* is introduced. At the same time, aside from direct trust evaluation between nodes, indirect trust evaluation of the to-be-evaluated nodes shall be collected from other neighboring nodes. This can be realized by collecting *r* and *s* information provided by other neighboring nodes. The statistic results of the existing forwarding behaviors of node *i* to neighboring node *j* are noted as $r_{ij}^{\text{old}}$ and $s_{ij}^{\text{old}}$. In the following time of Δ*t*, node *i* also records new $r_{ij}^{\Delta t}$ and $s_{ij}^{\Delta t}$ and receives the indirect trust evaluation *I*(*r*_*kj*_) and *I*(*s*_*kj*_) of other neighboring nodes. The newly successful forwarding times $r_{ij}^{\text{new}}$ and failed forwarding times $s_{ij}^{\text{new}}$ are calculated according to Formula (5) and Formula (6).

$$r_{ij}^{\text{new}}=\lambda r_{ij}^{\text{old}}+r_{ij}^{\Delta t}+\sum_{k\in \Omega ⋂k\neq j}I(r_{kj})$$ (5)

$$s_{ij}^{\text{new}}=\lambda s_{ij}^{\text{old}}+s_{ij}^{\Delta t}+\sum_{k\in \Omega ⋂k\neq j}I(s_{kj})$$ (6)

Where, Ω represents the neighboring nodes set of node *i*, *λ* ∈ [0, 1].

When the indirect trust evaluation is integrated into trust calculation, its reliability must be considered. The nodes of the third party that provides indirect trust evaluation may provide false information and deliberately defames good nodes or overstates malicious nodes. Formula (7) and Formula (8) respectively define *I*(*r*_*kj*_) and *I*(*s*_*kj*_). Indirect trust is endowed with certain weight to decrease the influence of false evaluation.

$$I(r_{kj})=\frac{2\;*\;r_{ik}\;*\;r_{kj}}{(s_{ik}+2)(r_{kj}+s_{kj}+2)+2\;*\;r_{ik}}$$ (7)

$$I(s_{kj})=\frac{2\;*\;r_{ik}\;*\;s_{kj}}{(s_{ik}+2)(r_{kj}+s_{kj}+2)+2\;*\;r_{ik}}$$ (8)

After node *i* calculates the trust of all neighboring nodes, neighboring nodes are divided into trusted nodes, common nodes and malicious nodes according Table 1.

**Table 1:** Trust division of neighboring nodes.

Trust Range	Trust Classification
0 < *T*_*ij*_ < *η*_2_	Malicious nodes
*η*_2_ ≤ *T*_*ij*_ < *η*_1_	Common nodes
*η*_1_ ≤ *T*_*ij*_ < 1	Trusted nodes

As the boundary value of malicious nodes and non-malicious nodes, the lower setting of *η*_2_ will influence the identification sensitivity of malicious nodes, making it possible for more malicious nodes to take part in routing forwarding; while the higher setting of *η*_2_ will reject some non-malicious nodes out of routing task, which will decrease the routing efficiency. As the boundary value of common nodes and trusted nodes, *η*_1_ can be used to distinguish the priority of the service request of these two nodes and stimulate the node collaboration in route and it shall be 0.5 more than the initial trust of nodes at least. The calculation of *η*_1_ and *η*_2_ are shown as Formula (9) and Formula (10).

$$\eta_{1}=\text{max}({\bar{T}}_{i},0.5)$$ (9)

$$\eta_{2}=\frac{1}{2}{\bar{T}}_{i}$$ (10)

Where, ${\bar{T}}_{i}$ represents the mean of all neighboring nodes’ trust values of node *i*. With the changing statistics of nodes participating in routing forwarding behaviors, ${\bar{T}}_{i}$ continuously changes, so do *η*_1_ and *η*_2_. Furthermore, the dynamic distinguishing boundaries of malicious nodes, common nodes and trusted nodes can be formed. The settings of *η*_1_ and *η*_2_ is relevant to the uncertainty of communication environment and specific application requirements.

### Trust-based routing model

In multi-hop CRNs, the neighboring nodes of communication must own the same available channel. The channels of each link on the path from source nodes to destination nodes vary a lot. The dynamic changes of nodes’ SOPs may lead to the reconstruction of current available routes. In this way, on-demand routing scheme is a good choice. At the same time, it is inevitable to face multi-channel switching problems in cognitive radio networks. Path delay becomes an important indicator of routing protocol design and routing optimization. In Literature [22], the cumulative delay along a route is derived by path delay and node delay, which represent the delay of spectrum assigned along the route and the delay of spectrum assigned around each node on the route, respectively. The effectiveness of candidate routes is evaluated by the cumulative delay. However, nodes' malicious behavior has not been considered. With nodes’ trust calculated in Section 2.3, TSRM (trust-based secure routing model) is proposed based on the routing scheme in Literature [22] to defend the selective forwarding attack of malicious nodes.

**Fig 2:**
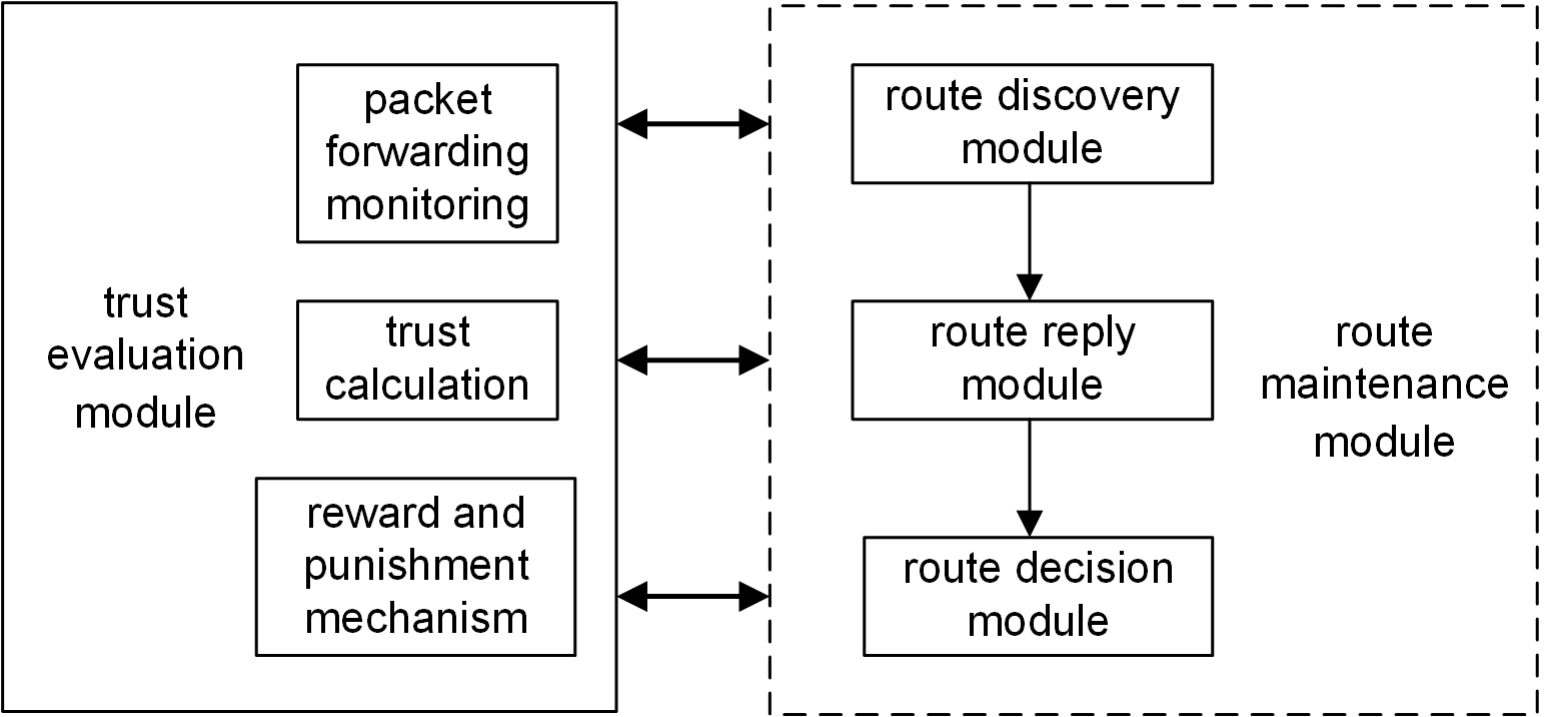
Trust-based safety routing model structure figure.

Shown as Fig 2, TSRM includes 5 modules, route discovery module, route reply module, route decision module, trust evaluation module and route maintenance module and each module contains several functions. Based on the function of trust evaluation module, route discovery module and route reply module can find all the available paths from source nodes to destination nodes. Based on path delay and trust, route decision module selects optimal route. Route maintenance module guarantees the real-time performance of information to decrease the unreachable probability of data packets.

#### Route discovery

When the data packet of node *i* needs to be sent to destination node *j* but there is no route reaching destination nodes in the routing list of node *i*, one route discovery progress will be triggered. The source node generates one RREQ, whose inside is packaged with SOPs information of node *i*. RREQ is sent to common nodes and trusted nodes of neighboring node *i* through common control channel to avoid malicious nodes to participate in data forwarding. After neighboring nodes receive SOPs, if SOPs in messages intersect with their SOPs, their SOPs are added and then RREQ is forwarded. If there is no intersection or message received from malicious nodes, this message shall be abandoned.

#### Route reply

When the destination node receives one RREQ, the optimal channel is selected from the intersection of SOPs in RREQ and its SOPs according to the minimum principle of path accumulation delay. The selected channel information is packaged into RREP which is sent according to the reverse transmission path of RREQ. After other middle nodes receive RREP, the processing is the same as the above. The only difference lies in that besides communication channel information of nodes and their neighboring nodes, RREP also packages the trust evaluation information of the next-hop’s neighboring nodes in forward path. At this time, the packaged trust evaluation information is transparent to the next-hop’s neighboring nodes. The path accumulation delay *D*_route,i_ of node *i* to destination node is shown as Formula (11).

$$D_{\text{route,i}}=DP_{i}+DN_{i}$$ (11)

Where, *DP*_*i*_ represents the path delay from node *i* to destination node and *DN*_*i*_ represents the sum of data transmission delay from node *i* to the destination node. The calculations of *DP*_*i*_ and *DN*_*i*_ can refer to Literature [22].

#### Route decision

After receiving n(*n* ≥ 1) RREP from source node *i*, there are multiple available routing paths. For available routing path *P*, its routing trust *T*_*P*_ is the minimum trust evaluation of the node in each link of this path to the next hop’s node, shown as Formula (12).

$$T_{P}=\text{min}(T_{ij})\;,\;(i,j)\in P\;,\;P=1,2\ldots n$$ (12)

For available routing path *P*, its path accumulation delay *D*_route,i_ from source node *i* to the destination node is noted as *D*_*P*_, shown as Formula (13).

$$D_{P}=D_{\text{route,i}}\;,\;P=1,2\ldots n$$ (13)

**Fig 3:**
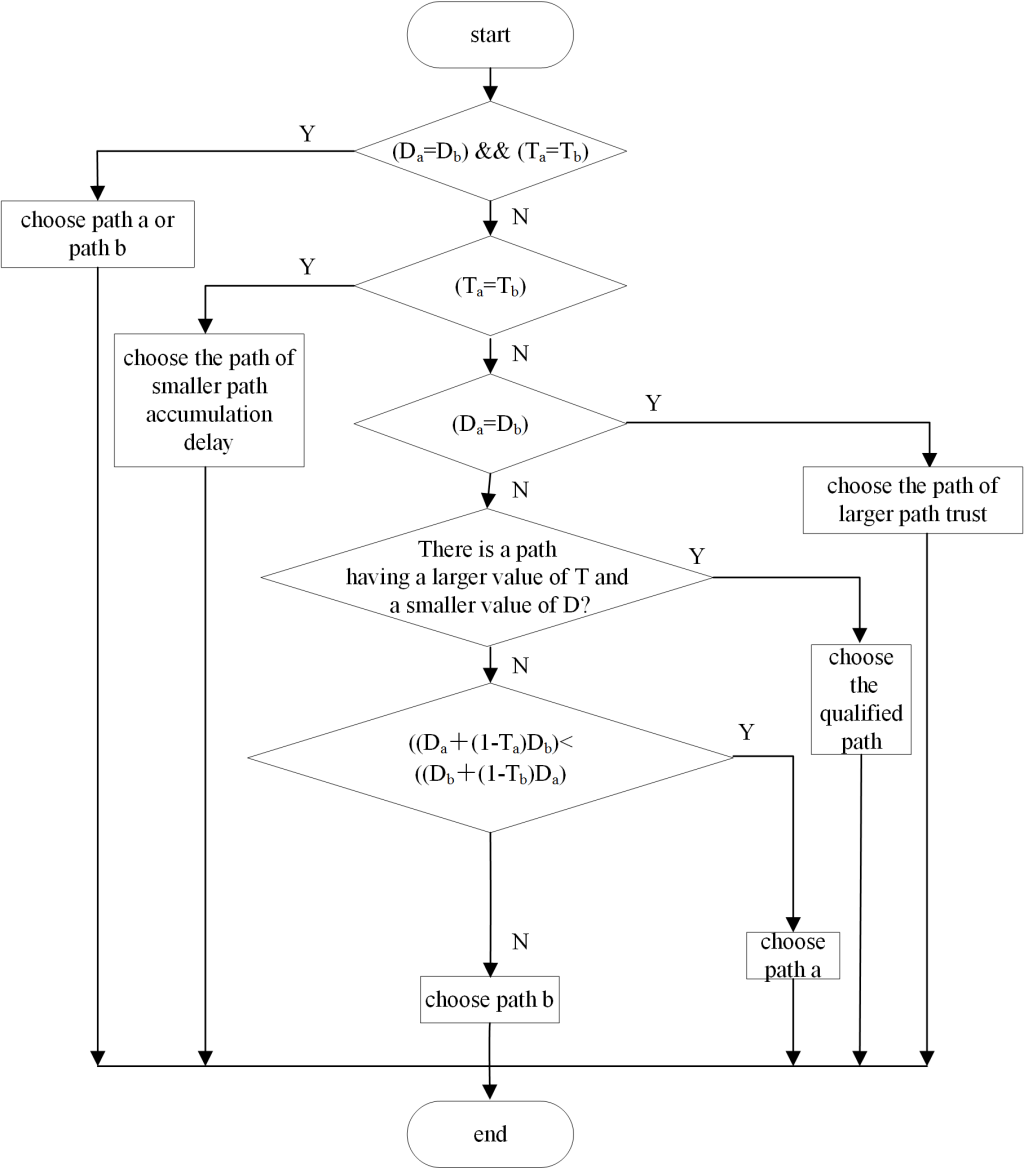
Route decision algorithm.

Suppose there are two available paths *a* and *b*. The corresponding path trust and path accumulation delay respectively are noted as *T*_*a*_, *D*_*a*_ and *T*_*b*_, *D*_*b*_. The route decision is carried out according to the algorithm flow chart in Fig 3.

If there are three available routing paths, all the possible values of path trusts and accumulation delays of them are detected. Except when there are two or more same path trusts and accumulation delays or all the accumulation delays are 0, there is always one available path better than the other two. Therefore, when there are n(*n* ≥ 3)available routing paths, we can choose one from two available routing paths according to the route decision algorithm in Fig 3 and discard the other one. Then the left paths are compared according to Algorithm 1 until the last one, namely, the optimal routing path is selected. At this time, data are transmitted.

#### Route maintenance

Middle nodes find that when the next hop’s link of current routing path is unavailable, wrong routing information is sent to source nodes to stop the data transmission of this path and restart route discovery or route decision. When middle nodes find the next hop’s neighboring nodes carrying out selective forwarding attack, the trust evaluation of this node decreases constantly. When malicious behaviors information is sent to other neighboring nodes and source nodes, the neighboring nodes update their trust evaluation and source nodes restart route discovery or route decision. At the same time, nodes periodically delete outdated route information in buffer memory for better management of buffer memory and update of routing information.

#### Reward and punishment mechanism

When nodes are in the selective forwarding attack, their failed forwarding times increases gradually in view of neighboring nodes, which makes the trust evaluation calculated from Section 2.3 constantly decrease until it falls into the scope of common nodes or malicious nodes. In the route discovery, malicious nodes will not receive RREQ and data forwarding will not pass malicious nodes. When nodes carry out route discovery for data transmission request, the priority of service request of common nodes is lower than the credible nodes. For malicious nodes, the service request is discarded. At the same time, for common nodes and malicious modes, when neighboring nodes monitor one failed forwarding, the failed forwarding times in the trust evaluations according to Section 2.3 are respectively noted as 1.5 and 2 times, which further aggravates the decreasing trust evaluation of non-trusted nodes. Therefore, common nodes and malicious nodes all are forced to honestly forward to improve the trust evaluation from neighboring nodes and further satisfy their own service requests. When non-trusted nodes fail to forward, heavier punishment of failed forwarding times conforms to the trust characteristics of slow growth and rapid decrease. However, how much the times increase with punishment level is not further explored due to limited space.

## Simulation Results and Analysis

To verify the validity of TSRM, NS2 [23] simulation is used to realize the TSRM and DORP [22] and ADOV[24] in multi-hop distributed CRN scene. 80 nodes are randomly distributed in the square region of 200*200 square units. The transmission range of each node is 15 units. SOPs are dynamic and can be divided into 6 available channels and the channel available probability obeys the distribution of 0~1. Each source node randomly selects destination nodes from other nodes, adopting constant bit rate data flow. In case of data forwarding, the proportion of malicious nodes in the network progressively increases of 5% and malicious nodes refuse forwarding or discard data packets with probability of 0.5, selective forwarding attack is further implemented. Each simulation is carried out many times. The data in the experimental analysis are the mean of repeated experimental results. Finally, the performances of AODV, DORP and TSRM under the selective forwarding attack are compared through network throughput and end-to-end delay. Among them, network throughput refers to the ratio of data packets that successively arrive at destination nodes to all sent data packets. End-to-end delay refers to the mean delay of data packets from source nodes to destination nodes.

### 1) Network throughput

**Fig 4:**
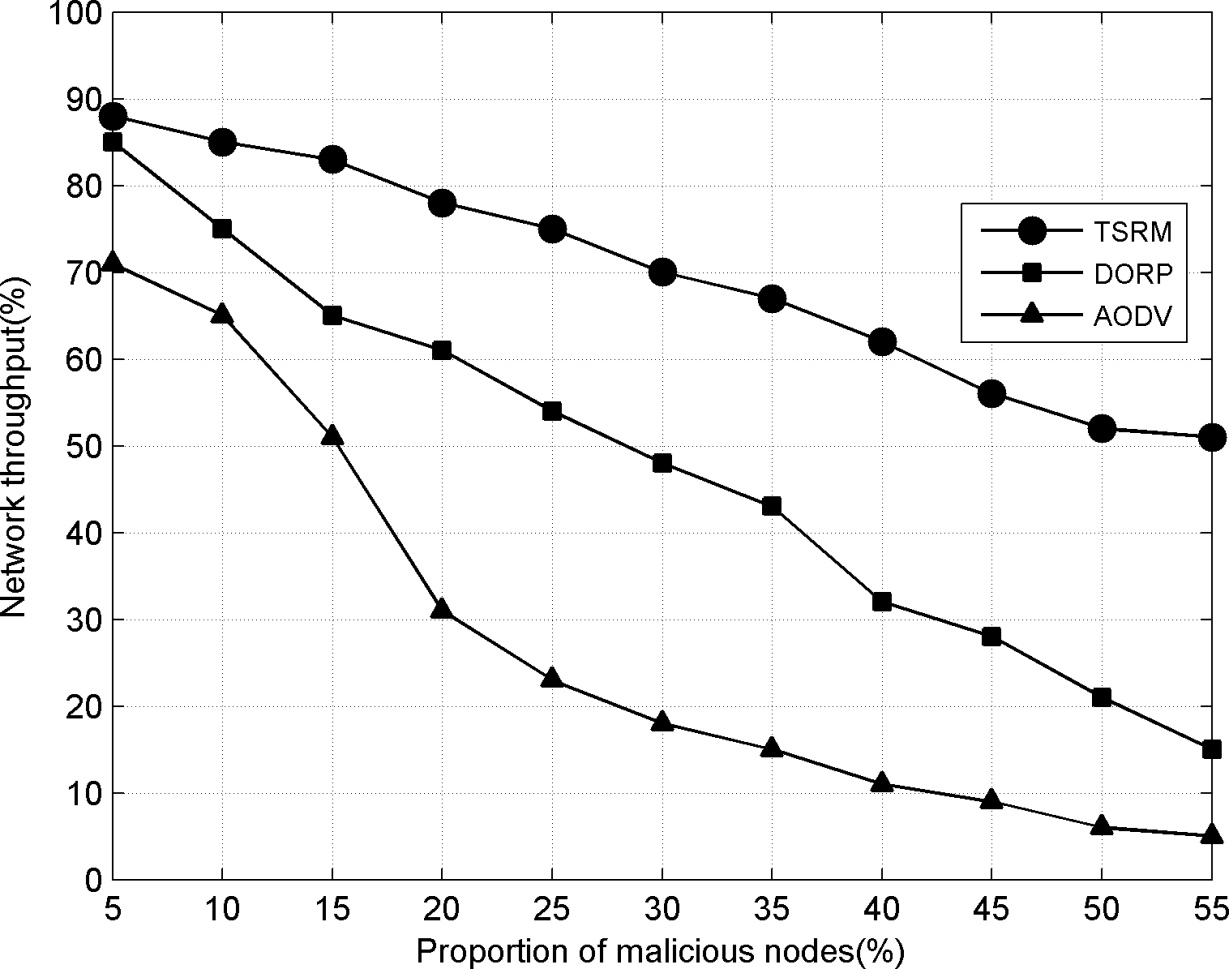
Network throughput.

It can be seen from Fig 4 that the introduction of trust mechanism enables the network throughput of TSRM to be better than those of DORP and AODV. Firstly, AODV does not consider the influence of the dynamic changes of nodes’ SOPs in CRNs on route. Routes are established according to on-demand distance vector algorithm. When data packets are forwarded, links usually break because there is no the same available channel between neighboring nodes. Based on AODV, DORP is improved and cross-layer design combines routing selection and spectrum allocation. However, under the selective forwarding attack, data packets passing malicious nodes are discarded in the forwarding route. In TSRM, the trust evaluation of neighboring nodes is established according to nodes' historical forwarding behaviors. In the route discovery and route decision, trust decreases the participation probability of malicious nodes in routing forward and improves the stability of links and implements reward and punishment mechanism to stimulate the cooperation of nodes. With the increasing proportion of malicious nodes, network throughput of DORP and AODV respectively decrease to 15% and 5%. The whole network basically is in an invalid state. Trust-based TSRM can effectively defend the influence of selective forwarding attack and the network throughput slowly decreases, maintaining above 50% all the time.

### 2) End-to-end delay

**Fig 5:**
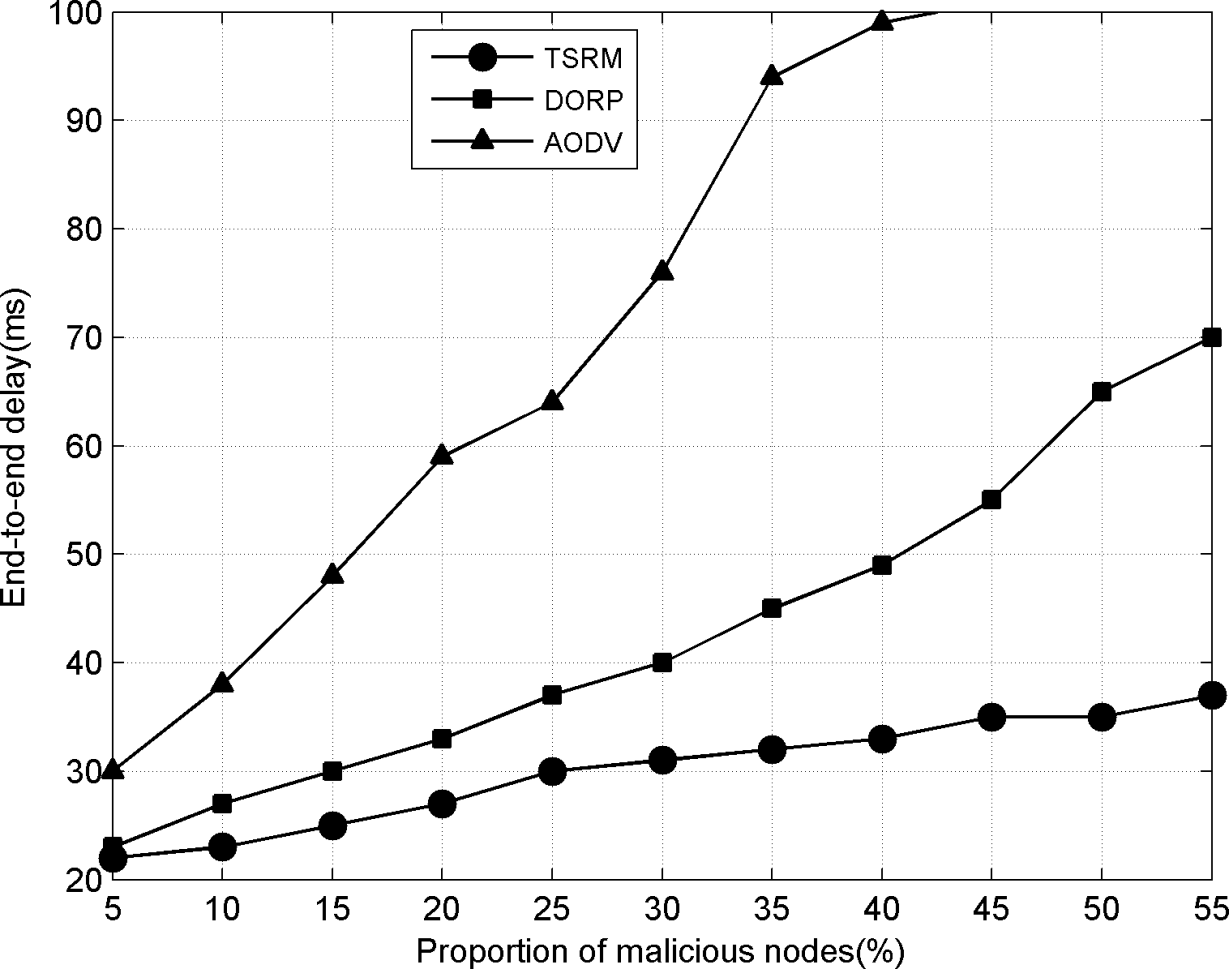
End-to-end delay.

In AODV, the determination of data forwarding route is based on the measurement of distance. DORP selects channels according to the minimum principle of path accumulating delay. Behaviors that malicious nodes refuse to forward or directly discard data packets always lead to failed forwarding of data packets under two schemes. The repeated failed reforwarding may further trigger the routing reconstruction process. Shown as Fig 5, when the proportion of malicious nodes in the network increases, the mean delay of data packets from source nodes to destination nodes is far higher than that of TRSM. Especially, the link interrupt caused by the dynamic changes of node SOPs in AODV aggravates the generation of reforwarding. When the proportion of malicious nodes is above 35%, end-to-end delay is beyond the normal condition of network tolerance. In TRSM, routes established on the basis of path trust and accumulating delay are relatively stable and maintain low end-to-end delay.

### 3) Node trust evaluation

**Fig 6:**
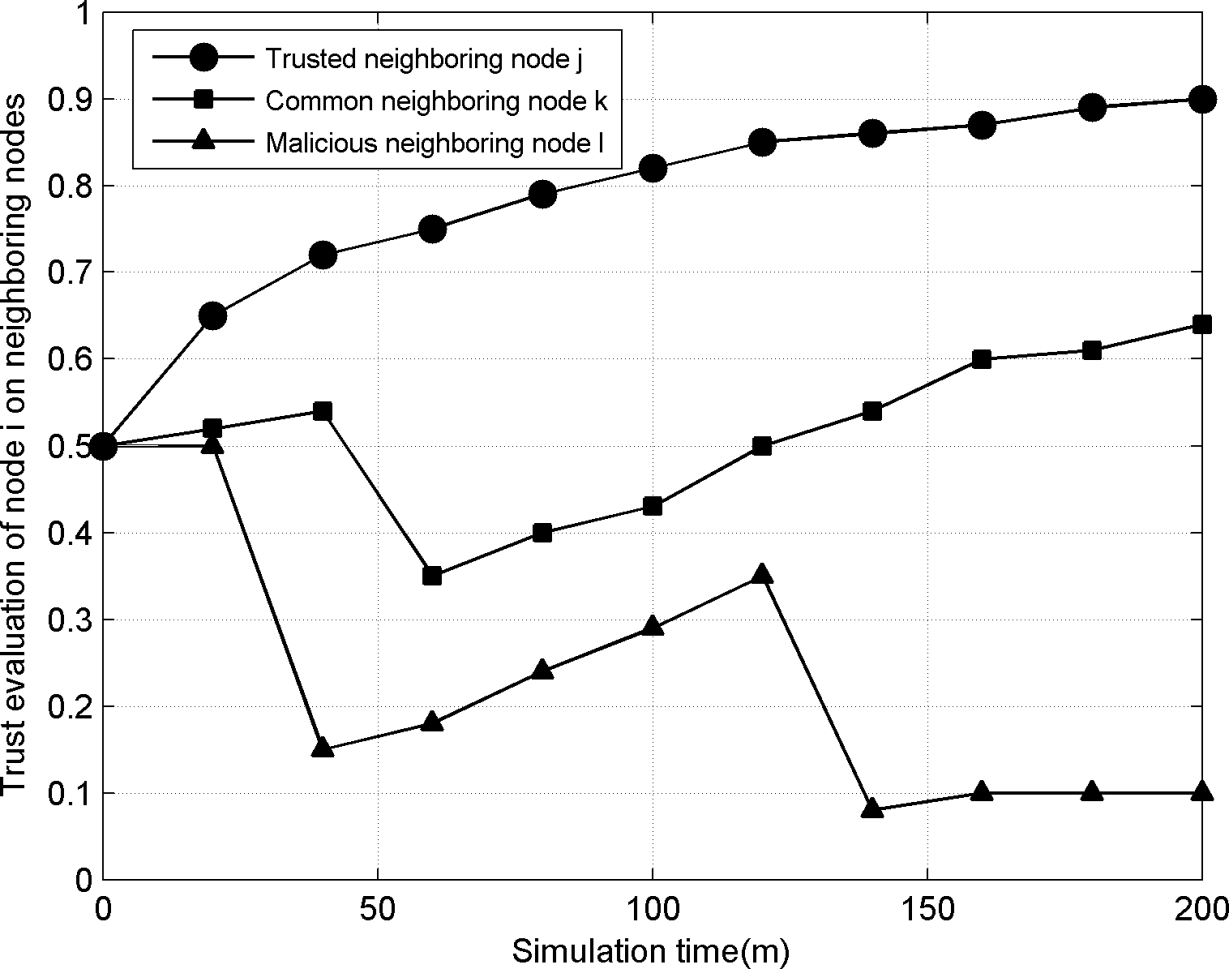
Trust changes of neighboring nodes.

Fig 6 shows the change process of trust evaluation of node *i* in TSRM on three neighboring nodes. Node *j* honestly forwards data packets from node *i* all the time and the trust evaluation of node *i* to it gradually increases. Finally Node *j* is listed in trusted nodes. Because of selective forwarding attack behaviors, the trust evaluation of node *i* based on reward and punishment mechanism to neighboring nodes *k* and *l* sharply decreases. Nodes *k* and *l* only can slowly improve the trust evaluation through honest forwarding. Due to the continuously honest forwarding of node *k*, the trust evaluation of node *i* increases later and node *k* is regarded as common nodes. Because node *l* has repeated attack behaviors after honest forwarding in certain time and the trust evaluation of node *i* decreases to certain threshold and node *l* is regarded as malicious nodes. In the trust-based routing model, malicious node *l* will not be chosen as the next-hop forwarding data of node *i* and its service request also cannot be satisfied. For the different forwarding behaviors of neighboring nodes, nodes will make different trust evaluations. At the same time, the malicious behaviors of untrusted nodes are punished severely so as to stimulate the node cooperation in routes.

**Fig 7:**
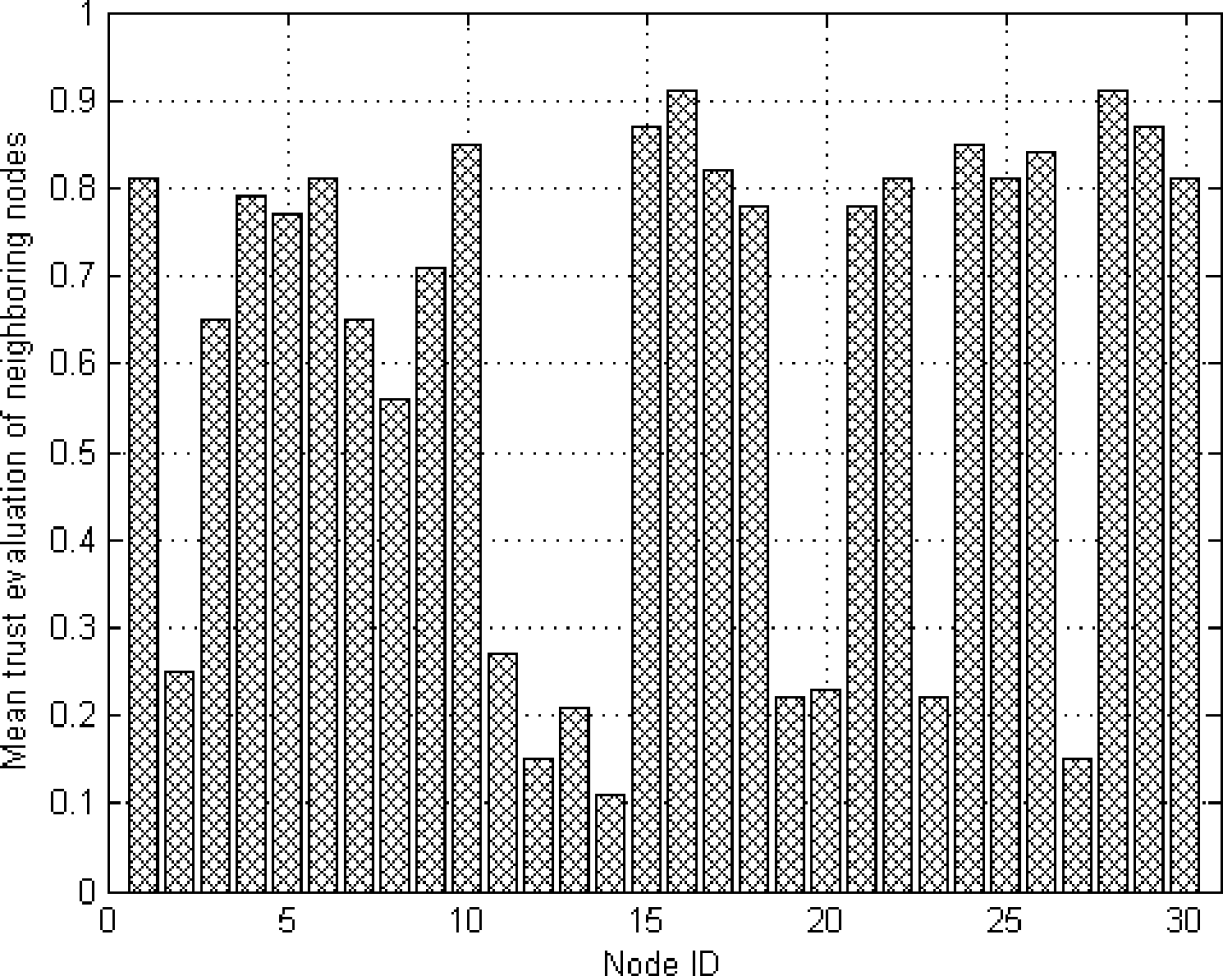
Mean trust evaluation of nodes.

Nodes 1–30 in networks are selected, including malicious node 2, node 11, node 12, node 13, node 14, node 19, node 20, node 23, node 27 and other non-malicious nodes. The mean trust evaluations of all the neighboring nodes to those nodes are observed. It can be seen from Fig 7 that malicious nodes own significantly low trust evaluation relative to common nodes and trusted nodes and it is verified that the model can effectively identify malicious nodes through trust evaluation and further provide basis for the following routing node selection.

## Conclusion

This paper puts forward trust-based secure routing model to defend the selective forwarding attack of malicious nodes. In the trust evaluation, the node forwarding behaviors are monitored and the beta reputation system is utilized to calculate the reliability of neighboring nodes. In the routing model, on the basis of the dynamic characteristic of nodes' SOPs in CRN route, delay, trust and other measurement indexes are integrated for route discovery, reply and decision. At the same time, reward and punishment mechanism is utilized to stimulate the node cooperation in routes and the simulation experiment verifies the validity of the model. This paper integrates the trust mechanism to CRN route scheme to defend the selective forwarding attack. The influence of SOPs dynamic changes on route establishment is guaranteed by route scheme but it fails to consider SOPs hiding and other selfish behaviors of nodes. The following work mainly includes, 1) further improving SOPs modeling analysis and reaction mechanism in trust models and testing and enhancing the validity of trust models in actual scenes; 2) introducing the trust mechanism into the other routing protocols in CRNs and constructing one more perfect secure model on the basis of different characteristics of CRN routing protocols and the common attacks to protocols, for example, using the game theory to take the selfish hiding behaviors of SOPs as one of factors of nodes' trust calculation; and 3) further studying the application of trust mechanism in CRN collaboration scene and utilizing trust to solve the route load balancing, data fusion and other problems in CRNs.
